# Prolific authors in ophthalmology and vision science

**DOI:** 10.5935/0004-2749.20210122

**Published:** 2025-08-21

**Authors:** Van C. Lansingh, Kristen A. Eckert, Armando E. Elizondo Quiroga

**Affiliations:** 1 Instituto Mexicano de Oftalmología, Queretaro, Queretaro, Mexico; 2 Help Me See, New York, NY, US; 3 Independent consultant, San Antonio Tlayacapan, Jalisco, Mexico; 4 Jalisco Secretary of Health, Guadalajara, Jalisco, Mexico

A report in 2016 revealed that 81 scientific authors have published more than 72 articles each year^(1)^. The pressure from the “publish or perish” principle advocated by research institutions and the financial incentives offered to researchers to publish in some countries have likely corrupted the author system and led to an increase in the number of “prolific” authors with an unrealistic number of articles^(1,2)^. Up to half of all authors have undeserved authorship^(3-7)^. Prolific authorship has not been studied in the field of ophthalmology and vision science; the average number of articles produced by researchers in these areas is unknown. In this review, we identified prolific authors in ophthalmology and vision science and estimated the general article rate.

A PubMed search using the search terms “ophthalmology” or “eye” or “vision” was conducted from January 1, 2019, through March 31, 2020. The filters “clinical trials” and “reviews” were applied. Titles were scanned to exclude articles unrelated to human ophthalmology/ vision science. Byline authors were extracted for analysis; the authors listed secondarily in groups, collaborations, and panels were excluded.

The 25 most prolific authors were identified. Their names were then searched without any filters for the study period to obtain their total number of publications. The top 10 most prolific authors were deidentified and listed in chronological order from the most prolific author (Author 1) to the prolific author with the least number of articles (Author 10). Their sex, number of affiliations, Global Burden of Disease super region^(8)^, and article type were summarized. The articles were categorized as randomized controlled trial (RCT), case report, laboratory science, other clinical science (including observational studies, case-control studies, and case series), review (including narrative reviews, systematic reviews, and meta-analyses), editorial, expert opinion, correspondence (including comments, replies, letters, and short communications), or other (including images, correction, and in memoriams). Articles not related to ophthalmology or vision science were categorized as “not ophthalmology”. The numbers of articles for which they were the first and corresponding authors and collaborators (not listed in the byline) were analyzed.

Each article of the most prolific authors was graded in accordance with the Oxford Centre for Evidence-Based Medicine scheme, based on a scale from 1 to 5, with 1 being the strongest level of evidence (systematic review of RCTs) and 5 beginning the weakest (mechanism-based reasoning)^(9)^. Descriptive statistics were used for all the analyses.

The PubMed search returned 5,579 articles, of which 1,958 (36%) were excluded. The 3,621 articles included were written by 13,239 authors. The mean number of articles per author was 1.3 (range: 1-37; median: 19). Twenty-seven authors (0.2%) had written more than 10 articles; their mean number of articles was 15.4. Unfiltered search results showed that their mean number of total articles was 57.6, which suggests that they pro duced 3.8 articles a month, or nearly 1 per week.

The top 10 prolific authors produced a mean 84.3 articles, or 5.6 articles a month (Table 1). Seven authors had multiple affiliations, and 9 authors were based in the high-income super region.

**Table 1:** Top 10 most prolific authors

Author no.	Female (Y/N)		No. of affiliations			GBD super region^A^		No. of articles, filtered		No. of articles, unfiltered
1	N		1			High-income		14		150
2	N		5			High-income (2 countries)		14		116
3	N		1			High-income		37		110
4	N		4			High-income		35		81
5	N		2			High-income		11		76
6	N		2			High-income		11		70
7	N		3			South Asia		13		64
8	N		3			High-income		14		59
9	Y		2			High-income		22		59
10	Y		1			High-income		15		58
Mean				2.4					18.6	84.3
SD				1.3					9.7	31.0
Median				2.0					14.0	73.0

GBD= Global Burden of Disease.

The mean numbers of articles for which each prolific author was listed as the first and corresponding author were 15.2 (20%) and 14.0 (18%), respectively (Table 2). They were listed as collaborators in 3% of all articles on average. The mean number of RCTs was 2.0 (3%; Table 3). The mean level of evidence in the articles by the 10 most prolific authors was 4.6 (SD: 0.47; Table 4).

**Table 2:** Characteristics of the top 10 most prolific authors

Author no.		No. of articles, unfiltered			No. (%) of authors listed as first author		No. (%) of authors listed as corresponding author			No. (%) of authors listed as collaborator
1		150			5 (3.3)		7 (4.7)			2 (1.3)
2		116			4 (3.4)		17 (14.7)			3 (2.6)
3		110			12 (10.9)		3 (2.7)			0 (0.0)
4		81			50 (61.7)		35 (43.2)			0 (0.0)
5		76			6 (7.9)		27 (35.5)			2 (2.6)
6		70			41 (58.6)		29 (41.4)			2 (2.9)
7		64			25 (39.1)		11 (17.2)			0 (0.0)
8		59			1 (1.7)		11 (18.6)			1 (2.9)
9		59			6 (10.2)		0 (0.0)			0 (0.0)
10		58			2 (3.4)		0 (0.0)			11 (19.0)
Mean		84.3			15.2 (20.0)		14.0 (17.8)			2.1 (3.1)
SD		31.0			17.5 (9.0)		12.6 (16.9)			3.3 (5.7)
Median		73.0			6.0 (23.8)		11.0 (15.9)			1.5 (2.0)

SD= standard deviation

**Table 3:** Number and types of articles of the top 10 most prolific authors based on unfiltered search

Author no.	No. of Articles	No. (%) RCTs	No. (%) of case reports	No. (%) of other clinical studies	No. (%) of laboratory studies	No. (%) of reviews	No. (%) of editorials	No. (%) of expert opinions	No. (%) of correspondences	No. (%) of articles of other categories	No. (%) of non-ophthalmology articles
1	150	2(1.3)	24 (16.0)	69 (46.0)	1 (0.7)	17(11.3)	4 (2.7)	2(1.3)	22 (14.7)	2(1.3)	0 (0.0)
2	116	3 (2.6)	1 (0.9)	70 (60.3)	0 (0.0)	32 (27.6)	4 (3.4)	0 (0.0)	3 (2.6)	2(1.7)	18(15.5)
3	110	0 (0.0)	1 (0.9)	1 (0.9)	0 (0.0)	106 (96.4)	2(1.8)	0 (0.0)	0 (0.0)	0 (0.0)	20(18.2)
4	81	1 (1.2)	7 (8.6)	10(12.3)	0 (0.0)	52 (64.2)	0 (0.0)	4 (4.9)	7 (8.6)	0 (0.0)	1 (1.2)
5	76	5 (6.6)	1 (1.3)	54 (71.1)	1 (1.3)	5 (6.6)	3 (3.9)	4 (5.3)	1 (1.3)	2 (2.6)	0 (0.0)
6	70	0 (0.0)	1 (1.4)	8 (11.4)	0 (0.0)	20 (28.6)	1 (1.4)	1 (1.4)	33 (47.1)	1 (1.4)	3 (4.3)
7	64	0 (0.0)	6 (9.4)	1 (1.6)	0 (0.0)	51 (79.7)	0 (0.0)	0 (0.0)	4 (6.3)	2(3.1)	0 (0.0)
8	59	3(5.1)	7(11.9)	23 (39.0)	9(15.3)	9(15.3)	0 (0.0)	0 (0.0)	5 (8.5)	2 (3.4)	0 (0.0)
9	59	1 (1.7)	3(5.1)	6(10.2)	0 (0.0)	44 (74.6)	0 (0.0)	4 (6.8)	1 (1.7)	0 (0.0)	1 (1.7)
10	58	5 (8.6)	2 (3.4)	34 (58.6)	0 (0.0)	10(17.2)	0 (0.0)	2 (3.4)	3 (5.2)	2 (3.4)	0 (0.0)
Mean	84.3	2.0 (2.7)	5.3 (5.9)	27.6(31.1)	1.1 (1.7)	34.6 (42.1)	1.4 (1.3)	1.7 (2.3)	7.9 (9.6)	1.3 (1.7)	4.4 (4.3)
SD	31.0	1.9 (3.0)	7.0 (5.3)	27.6 (26.8)	2.8 (4.8)	30.6 (33.1)	1.7 (1.6)	1.8 (2.6)	10.8 (13.9)	0.9 (1.4)	7.8 (6.8)
Median	73.0	1.5 (1.5)	2.5 (4.3)	16(25.7)	0.0 (0.0)	26.0 (28.1)	0.5 (0.7)	1.5 (1.4)	3.5 (5.7)	2.0 (1.6)	1.0 (1.5)

RCT= randomized controlled trial; SD= standard deviation.

**Table 4:** Summary of the levels of evidence of articles authored by the top 10 most prolific authors based on unfiltered search

Author no.	No. articles	Mean (SD) level of evidence	No. (%) of level 5 articles	No. (%) of level 4 articles	No. (%) of level 3 articles	No. (%) of level 2 articles	No. (%) of level 1 articles
1	150	4.5 (0.58)	75 (50.0)	73 (48.6)	0 (0.0)	2 (1.3)	0 (0.0)
2	116	3.8 (0.78)	18 (15.5)	65 (56)	25 (21.6)	2 (6.9)	0 (0.0)
3	110	5 (0.09)	109 (99.1)	1 (.09)	0 (0.0)	0 (0.0)	0 (0.0)
4	81	4.9 (0.38)	71 (87.7)	9 (11.1)	1 (1.2)	0 (0.0)	0 (0.0)
5	76	4.1 (0.6)	15 (19.7)	52 (68.4)	8 (10.5)	1 (1.3)	0 (0.0)
6	70	4.9 (0.49)	63 (90)	5 (7.1)	1 (1.4)	1 (1.4)	0 (0.0)
7	64	5 (0.12)	63 (98.4)	1 (1.6)	0 (0.0)	0 (0.0)	0 (0.0)
8	59	4.5 (0.57)	34 (57.6)	23 (39)	2 (3.4)	0 (0.0)	0 (0.0)
9	59	4.9 (0.39)	52 (88.1)	6 (10.2)	1 (1.7)	0 (0.0)	0 (0.0)
10	58	3.9 (0.93)	13 (22.4)	32 (55.2)	5 (8.6)	8 (13.8)	0 (0.0)

SD= standard deviation

By using PubMed filters to capture original research studies on ophthalmology and vision science, only 0.2% of authors (27/13,239) had more than 10 articles published. While our search was not comprehensive of all articles published by ophthalmologists and included non-ophthalmologist authors, the use of the broad search terms “ophthalmology” and “vision science” was expected to capture a fair representation of authors. The mean number of articles per author was 1.3 in the 15-month period, which suggests that the annual number of original research articles per author in ophthalmology and vision science is only 1.

The unfiltered search for prolific authors revealed that they produced nearly 1 article per week. The 10 most prolific authors generated more than 1 article per week, with the most prolific author having more than 2 articles per week and nearly 3 times as many articles as the 10th most prolific author (150 vs 58 articles). Three authors had more than 10 articles published per month, which suggests that only a few authors in ophthalmology and vision science have an unrealistic article rate. Most prolific authors (70%) had multiple affiliations, allowing for increased collaboration.

All prolific authors except for one were from a high-income super region. The lack of representation from low-to-middle income countries is not surprising, because these authors often face an English language barrier, limited financial resources and support to cover the research expense and high publishing fees, and a discriminating peer-review process^(10)^.

The mean level of evidence in the articles by the most prolific authors was nearly 5, the lowest level of evidence (Table 4). None of the articles were level 1, because the systematic reviews by the prolific authors were all observational studies (not just RCTs); thus, the articles were graded down to level 2. Only few RCTs were published (Table 3). Study design flaws related to poor power calculation and high attrition rates and/or poor reporting led to several RCTs being graded down one level. The prolific authors most frequently produced reviews (mean, 42% of all articles). The highest mean level of evidence of 3.8 was produced by Author 2. Author 1, who had 150 articles with a mean level of evidence of 4.5, produced 24 case reports and 22 correspondences. All 110 articles by Author 3 had a level of evidence of 5, except for one (level 4), of which 18.2% were not related to ophthalmology or vision science.

Only few prolific authors were found in the field of ophthalmology and vision science. Our study findings suggest that authors in ophthalmology and vision science produce one original research article per year, which may be a considerably higher number among ophthalmologists working in rigorous, academic research settings. Our data demonstrate that prolific authorship in ophthalmology and vision science does not necessarily translate to strong evidence-based medicine. If authors were consistently evaluated on quality rather than quantity, the resulting evidence base would be much stronger, and prolific authors would be less incentivized.
